# Procedural Training in Acute Care: A Prospective Study of Learning Intubation Highlighting a Novel Method

**DOI:** 10.1155/emmi/9940852

**Published:** 2026-01-05

**Authors:** Austin Milton, Rusha Patel, Lurdes Queimado, Price Sonkarley, Edward Kosik, Marvin Williams, Michael Anderson, Alexis Patsias, Michael Clampitt, Rachel Hardy, Nilesh R. Vasan

**Affiliations:** ^1^ Department of Internal Medicine, University of Utah Health, Salt Lake City, Utah, USA; ^2^ Department of Otolaryngology, University of Oklahoma Health Sciences Center, Oklahoma City, Oklahoma, USA, ouhsc.edu; ^3^ Department of Anesthesiology, University of Oklahoma Health Sciences Center, Oklahoma City, Oklahoma, USA, ouhsc.edu; ^4^ Department of Obstetrics and Gynecology, University of Oklahoma Health Sciences Center, Oklahoma City, Oklahoma, USA, ouhsc.edu; ^5^ Department of Biostatistics and Epidemiology, University of Oklahoma Health Sciences Center, Oklahoma City, Oklahoma, USA, ouhsc.edu; ^6^ College of Osteopathic Medicine, Oklahoma State University, Tulsa, Oklahoma, USA, okstate.edu

**Keywords:** airway management, education, endotracheal intubation, simulation training

## Abstract

**Study Hypothesis:**

The researchers compared average intubation times between four different devices and hypothesized that a novel laryngoscope based on an enhancement of the rigid anterior commissure laryngoscope would produce faster times to intubation compared to a Macintosh blade with a bougie among inexperienced users on the difficult airway simulation.

**Methods:**

Participants were stratified into novice, intermediate, and advanced skill levels. Each group first performed intubation on a manikin airway without modifications (“easy” airway)—using each of four devices (novel laryngoscope, Macintosh alone, Macintosh with bougie, and GlideScope) in random order—followed by the same technique on a manikin with modifications to mimic a “difficult” airway. Devices requiring the use of a bougie utilize a Seldinger technique. The primary outcome measure was the time taken to inflate the manikin’s lungs with the bag ventilator.

**Results:**

Ninety‐eight participants were recruited and grouped according to their self‐reported experience level: 41 novices, 39 intermediate, and 18 experts. The novel laryngoscope with gum elastic bougie (GEB) led to quicker intubation times (mean 32.0 s) compared with the Macintosh with GEB (mean 37.5 s) among the novice and intermediate groups on the difficult airway (*p* < 0.05). The methods that utilized a bougie (Macintosh blade with a GEB and Novel Laryngoscope with GEB) led to slower intubation times than the methods not utilizing a bougie (Macintosh blade and GlideScope).

**Conclusions:**

In summary, the Seldinger technique is an important skill for those who perform endotracheal intubations (ETIs), even infrequently or in nonideal settings. The novel laryngoscope may be a helpful option to attain ETI with the reliability of the Seldinger technique and a consistently short ETI interval.

## 1. Introduction

Intubation is a life‐saving skill that can be difficult to learn [1, 2] and retain [3, 4]. Conventional intubation methods include the use of a Macintosh, Miller, or video‐assisted laryngoscope—each with its own limitations. All three can be used with or without the assistance of a bougie or stylet. It has been shown that the use of a bougie (tracheal introducer) compared with an endotracheal tube (ETT) and stylet (malleable metal rod) may result in significantly higher first‐attempt success among patients undergoing emergency endotracheal intubation (ETI) [5]. Video‐assisted laryngoscopes are typically used instead of Macintosh or Miller blades during difficult airway intubation [6]. Video‐assisted intubation systems have been shown to produce a better view of the glottis and a higher success rate in difficult intubations than direct intubation with a Macintosh blade. [7–11]. Video laryngoscopy has some limitations, including being cost prohibitive and not universally available. Studies have shown intubation using video laryngoscopy to be slower than direct laryngoscopy, particularly among experienced users [8, 12–15] (GlideScope, Pentax, McGrath, Viewmax); however, this finding is sometimes reversed when measuring intubation by novice users (GlideScope, Pentax) [9, 16]. To the authors’ knowledge, there has not been a study comparing ETI times of users at different experience levels with different laryngoscopy methods using easy and difficult simulation models.

Video laryngoscopy devices tend to be expensive, making them less available in situations where reliable intubation is critical—typically outside the operating room or hospital [12]. The camera and light may also be obscured by blood in the field in situations where other methods would maintain vision [17]. First responders are often forced to rely on the various sizes of Macintosh and Miller blades, insertion of supraglottic devices, or resort to a cricothyroidotomy if they are unsuccessful with intubation, which has a high rate of complication [18, 19]. This study tested a prototype laryngoscope referred to herein as the novel laryngoscope (NL) and related to a product marketed as the Vie Scope [20]. It is based on an enhancement of the rigid anterior commissure laryngoscope commonly used by ENT surgeons and may have value for inexperienced users and in settings where video laryngoscopy is not available [21]. The rigid anterior laryngoscope, compared to conventional laryngoscopes, has a shorter, tubular blade with a light source designed to focus anteriorly and provide room for instrumentation. Enhancements offered by the NL include a clear tube that optimizes the visual area, a built‐in ring of LEDs, and an ergonomic design.

The objective of this study was to compare the average intubation times between four different devices used by individuals of varying experience levels to determine if the NL results in faster intubation times on difficult airways. The researchers hypothesized that the NL would produce shorter times to intubation compared to a Macintosh blade with a bougie among inexperienced users on the difficult airway simulation. A secondary objective was to compare how improvement in intubation time differed among the different techniques. The researchers hypothesized that participants would improve faster with the NL than with a Macintosh blade with a bougie.

## 2. Materials and Methods

### 2.1. Participants

This prospective study was conducted between August 2015 and May 2017 and was performed with Institutional Review Board approval (IRB number 5768). Adult participants were recruited using flyers posted within the University of Oklahoma Health Sciences Center Hospital and Medical School. The cohort was stratified according to level of experience with intubation. Participants self‐reported their experience level with each of the four methods described below and with intubation in general using a Likert‐type scale ranging from 0 to 3—with 3 being expert and 0 being inexperienced. Those who reported 3 (expert) on three or more intubation methods were stratified in the experienced group. Those who reported all 0’s were stratified in the novice group. Participants with experience levels between these two groups were put in an intermediate group. This grouping used the natural learning curve to minimize bias by setting a high bar for the expert group (which was naturally filled with attendings, senior anesthesiology residents, etc.) and a low bar for novices (with essentially no experience). None of the participants had ever used the NL before this study.

### 2.2. Protocol

**Figure 1:**
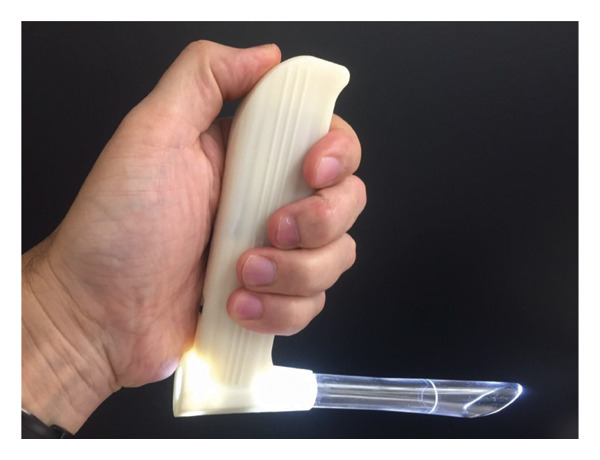
“Difficult” airway scenario in a manikin, modified with packing within the floor of the mouth, a neck collar, and taping of the mouth to replicate trismus.

The four laryngoscopy methods compared in the trial include a Macintosh 3 blade, with and without a bougie; a GlideScope Advanced Video Laryngoscope with a stylet (Verathon, Bothell, Washington, USA); and the NL with a bougie. A size 7.0 ETT was used for all four techniques and in both easy and difficult airway scenarios. For the two methods that did not use a bougie, a soft stylet was used inside the ETT. For the two methods that utilized a bougie, this study did not provide an assistant to pass a bougie to the participant, which meant participants may choose to look away from the scope to obtain it from the table. AirSim Advance manikins (TruCorp, Belfast, Ireland) were used for both the easy and difficult simulation airways. The “easy” airway scenario used a manikin with the tongue deflated and unfixed neck for increased extension. The “difficult” airway scenario had the tongue fully inflated, packing inserted underneath the floor of the mouth, and the neck fixed with a hard cervical collar (Figure 1). Tape was also placed around the manikin head and jaw to allow only 2.5 cm of mouth opening.

Study participants were provided with standardized instructions for each intubation device by showing a video demonstrating each method’s correct technique. The videos were each around 5 min and were reviewed for accuracy beforehand by an experienced member of the research team (EK) to ensure consistency with relevant guidelines and best practices. Videos were obtained from third‐party medical education sources, except for the NL video, which was recorded by the study team. Each study participant was then allowed time to familiarize themselves with each process utilizing an airway manikin. This consisted of 5–20 min spent at their own discretion without guidance from research personnel. At no point during the study were participants provided with formal training for any intubation techniques.

**Figure 2:**
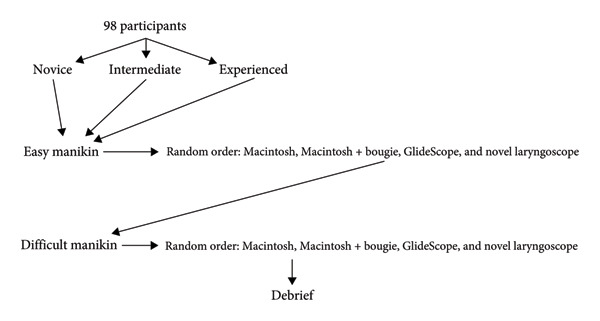
Flowsheet of intubation attempt sequencing.

Study participants were allowed three attempts with each of the four intubation techniques, beginning with the manikin with a normal (easy) airway and then moving on to the difficult airway (Figure 2). This meant every participant had a total of 24 intubation attempts in the 8 scenarios with 3 attempts for each. The trial was randomized by giving each participant a random number correlated with a unique order in which each technique would be tested on the study manikins.

### 2.3. NL

**Figure 3:**
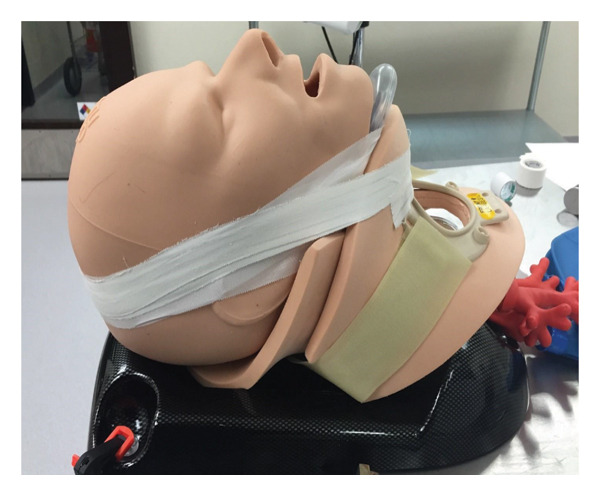
The novel laryngoscope with a clear enclosed circular tube and proximal light‐emitting diode light source. The anterior commissure design, with its angled, flared‐open end, gives the user a view of the vocal cords with a straight line of sight, which allows guidance of the bougie past the glottis and into the trachea under direct vision.

The NL consists of a clear polycarbonate plastic tube measuring 14 cm in length from the handle to the tube’s tip. It has a built‐in, battery‐powered, ring LED light source at the proximal end of the tube (Figure 3) [22]. The ergonomic handle is made of 3D‐printed plastic with ridges to suit a variety of hand shapes and sizes. The NL was developed for the “nonideal setting,” (where fiberoptic or video laryngoscopes may be unavailable and patients may have a difficult or traumatic airway). It allows gentle pressure against the upper teeth and gums, which enables the scope to obtain a laryngeal axis more easily. While conventional anterior commissure laryngoscopy is often performed in the operating room with a dental guard to mitigate damage from incidental incisor contact when attempting to visualize the glottic opening, the plastic surfaces of the NL reduce contact pressure on the incisors, so it may be used without a dental guard during simulation [23]. The commercially available Vie Scope incorporates a built‐in rubber tooth guard to avoid dental injury. In contrast, other methods may require significant pressure against the tongue to avoid contacting the teeth by metal blades and are associated with a higher rate of dental damage [24]. Although the clear tube provides some panoramic visibility, the aperture has a more focused view of the larynx compared to typical video laryngoscopy. Unlike the curved Macintosh blade, which may align oral, pharyngeal, and laryngeal axes by lifting the tongue base, the cylindrical blade of the NL has a minimally curved beveled end and relies more on direct advancement to expose the glottis by elevating the epiglottis with the tip of the scope. In a patient without cervical spine precautions, head extension and external laryngeal manipulation (BURP) may align the 3 axes. The NL bypasses some of this limitation with a direct “tube‐to‐larynx” direct line of sight, which can be performed in a neutral neck position. There is now a pediatric version that is more suitable for smaller oral apertures.

The NL method utilizes a Seldinger technique, which requires the use of a bougie. The bougie is passed through the scope’s lumen under direct visualization until it passes beyond the vocal cords into the trachea. The NL is removed with the bougie in place. An ETT is then passed over the bougie into the trachea before the bougie is removed [25]. The NL acts as a bougie introducer. This differs from a Macintosh blade or video laryngoscope in that the enclosed tube directs the bougie and may assist with passing accurate placement.

### 2.4. Measurements

Demographic data, including participants’ occupation/education level, handedness, and gender, were recorded. The primary outcome measure was the time taken to inflate the manikin’s lungs with a bag ventilator. Timing began when the laryngoscope, ETT, or any adjunct was first handled by the participant and ended when the participant inflated the manikin’s lungs with the bag ventilator. Participants were required to declare when they had a “good view” of the vocal folds and were ready to place the ETT or bougie—and this time was also recorded. In the event of an unsuccessful intubation, the reason was documented. Esophageal intubations and attempts stopped due to a broken prototype were recorded separately. Failure to pass the ETT through the vocal cords without esophageal intubation was recorded as a failed attempt. The protocol did not differentiate between endotracheal and bronchial intubation or include confirmation of ETT insertion depth, partly due to the style of the manikin. Attempts taking more than 90 s were stopped by the researchers and documented as failed. The use of a 90‐s cutoff in this study reflected the early‐stage learning environment. Duration of safe apnea is very patient‐specific, and there is no definite cutoff. This is especially true in the context of modern preoxygenation and apneic oxygenation. Attempts aborted by the participant for any reason besides a broken device were also documented as failed attempts.

### 2.5. Statistical Analysis

Two‐sided, paired *T*‐tests were performed using Microsoft Excel to assess if there is a difference in average times to successful intubation between the different methods. The times to successful, complete intubation were averaged separately and analyzed for each difficulty level, attempt number (first, second, or third), and experience group. Two‐sided, paired *T*‐tests were also performed for the secondary objective to compare the percent improvement with the Macintosh blade with a bougie to each other device for every skill level. A *p* value of 0.05 was considered the threshold for significance.

## 3. Results

### 3.1. Characteristics of Study Subjects

Ninety‐eight participants were recruited: 41 novices, 39 intermediates, and 18 experts. Table 1 demonstrates the demographic data of the participants. Demographic data are reported to monitor diversity in the study population, and were not analyzed against intubation time. Intubation time and success do not differ based on gender [26]. Left‐handedness may affect procedural training; however, research in this area is often neglected [27].

**Table 1:** Study participant demographics.

	Novice (*n*)	Intermediate (*n*)	Expert (*n*)	Total (*n*)
Study population				
Medical student	34	15	0	49
Resident	1	22	11	34
Physician or CRNA	0	1	6	7
Other^∗^	6	1	1	8
Gender				
Male	28	27	15	70
Female	13	12	3	28
Handedness				
Left	2	5	1	8
Right	39	34	17	90

Abbreviation: CRNA = certified registered nurse anesthetist.

^∗^Included nurse, social work, barista, and unreported.

### 3.2. Main Results

**Figure 4:**
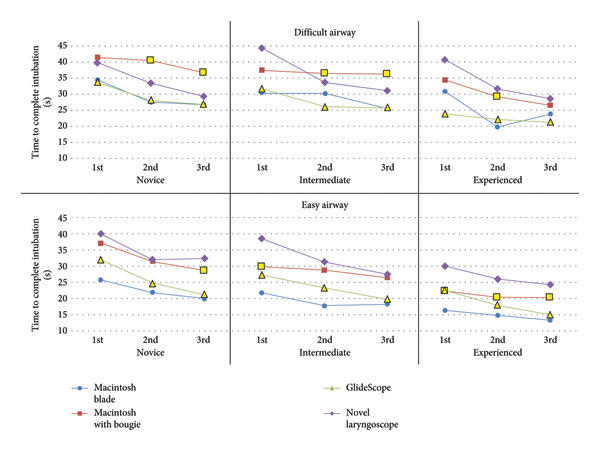
Change in total intubation time across three attempts. ^∗^For the GlideScope and Macintosh blade with a bougie, markers that are yellow with a black outline indicate a statistically significant difference compared to intubation time using the novel laryngoscope, as determined by a two‐sided, paired *T*‐test.

The NL led to quicker intubations (mean 32.0 s) compared with the Macintosh with bougie (mean 37.5 s) among the novice and intermediate groups on their second and third attempts with the difficult airway (*p* < 0.05). The NL led to longer intubation times (mean 31.3 s) compared with the Macintosh with a bougie (mean 27.2 s) for the easy airway for all three experience levels (*p* < 0.05). Both methods that utilized a bougie (Macintosh blade with a bougie and NL) resulted in longer intubation times than the methods not utilizing a bougie (Macintosh blade and GlideScope with stylet) for every skill level on both the easy and difficult airway (*p* < 0.05). The Macintosh blade without bougie led to faster intubations than the GlideScope for all three skill levels on the easy airway (*p* < 0.05) and did not show a statistical difference from the GlideScope for any group on the difficult airway (*p* > 0.05). Figure 4 illustrates key differences between the NL and other methods. See Tables 2 and 3​ in Supplemental Data for intubation times, standard deviations, and all *p-*values.

**Table 2:** Intubation time data: easy airway.

	**Macintosh blade**			**Macintosh w/bougie**			**GlideScope**			**Novel laryngoscope**		
**Attempt**	**1st**	**2nd**	**3rd**	**1st**	**2nd**	**3rd**	**1st**	**2nd**	**3rd**	**1st**	**2nd**	**3rd**

	**Novice participants**											
*n*	40	40	41	40	41	40	41	41	41	39	36	38
Mean time (s)	25.7	21.7	20.0	37.0	31.4	28.4	32.0	24.7	21.3	40.1	32.0	32.4
SD	11.3	8.9	7.3	14.3	9.4	11.9	13.5	8.1	7.4	17.6	13.8	13.2

*T*‐test: Mac. w/Bougie	0.00	0.00	0.00				0.04	0.00	0.00	0.14	0.44	0.04
*T*‐test: GlideScope	0.01	0.04	0.17	0.04	0.00	0.00				0.00	0.00	0.00

	**Intermediate participants**											
*n*	39	39	38	36	38	39	37	38	38	36	37	38
Mean time (s)	21.9	17.9	18.4	29.7	28.8	26.6	27.3	23.2	19.7	38.6	31.3	27.3
SD	8.5	4.9	6.0	13.0	11.2	8.5	13.5	11.0	8.7	17.8	13.6	8.5

*T*‐test: Mac. w/Bougie	0.00	0.00	0.00				0.09	0.00	0.00	0.01	0.11	0.26
*T*‐test: GlideScope	0.01	0.00	0.13	0.09	0.00	0.00				0.00	0.00	0.00

	**Experienced participants**											
*n*	18	18	18	18	18	18	18	18	18	18	18	18
Mean time (s)	16.3	14.8	13.3	22.3	20.2	20.2	22.6	18.0	15.1	30.0	26.1	24.2
SD	4.6	4.9	3.6	5.2	5.9	6.8	13.6	7.0	4.7	13.1	9.9	6.0

*T*‐test: Mac. w/Bougie	0.00	0.00	0.00				0.45	0.046	0.00	0.01	0.00	0.00
*T*‐test: GlideScope	0.02	0.00	0.04	0.45	0.046	0.00				0.02	0.00	0.00

**Table 3:** Intubation time data: difficult airway.

	**Macintosh blade**			**Macintosh w/Bougie**			**GlideScope**			**Novel laryngoscope**		
**Attempt**	**1st**	**2nd**	**3rd**	**1st**	**2nd**	**3rd**	**1st**	**2nd**	**3rd**	**1st**	**2nd**	**3rd**

	**Novice participants**											
*n*	36	36	34	27	33	35	35	38	41	36	38	36
Mean time (s)	34.5	27.6	26.6	41.6	40.5	36.7	33.8	28.1	26.9	39.8	33.5	29.4
SD	17.5	14.2	15.7	24.8	23.4	19.8	18.3	14.2	13.9	19.3	14.0	12.8

*T*‐test: Mac. w/Bougie	0.09	0.00	0.01				0.01	0.00	0.00	0.06	0.02	0.01
*T*‐test: GlideScope	0.27	0.36	0.27	0.01	0.00	0.00				0.03	0.01	0.04

	**Intermediate participants**											
*n*	37	32	34	28	33	32	39	38	38	31	32	33
Mean time (s)	30.4	30.1	25.6	37.5	36.5	36.2	31.8	26.1	25.9	44.4	33.7	31.3
SD	14.8	19.6	15.3	21.1	19.6	21.5	15.7	11.9	12.9	24.2	18.1	14.8

*T*‐test: Mac. w/Bougie	0.00	0.06	0.00				0.06	0.00	0.00	0.27	0.01	0.04
*T*‐test: GlideScope	0.41	0.09	0.45	0.06	0.00	0.00				0.00	0.00	0.00

	**Experienced participants**											
*n*	18	18	18	18	16	18	18	18	18	17	18	17
Mean time (s)	31.0	19.9	24.0	34.6	29.1	26.6	24.0	22.3	21.4	40.7	31.7	28.7
SD	19.4	8.5	8.7	14.4	15.9	10.6	13.3	7.9	10.2	21.2	10.2	10.1

*T*‐test: Mac. w/Bougie	0.26	0.00	0.15				0.02	0.03	0.051	0.17	0.049	0.28
*T*‐test: GlideScope	0.11	0.15	0.19	0.02	0.03	0.051				0.01	0.00	0.02

### 3.3. Secondary Objective: Comparison of Improvements in Intubation Times

On the difficult airway, the only significant difference in percent improvement between any of the devices was the NL had a higher rate of improvement (29%) in intubation time than the Macintosh blade with a bougie (4%) among intermediate participants (*p* = 0.037). Intermediate participants on the easy airway improved quicker in intubation times with the NL (29%) than with the Macintosh blade with a bougie (11%; *p* = 0.001). Experienced participants did not show any statistically significant differences on either difficulty level. Table 4 contains all percent improvement calculations and *p* values.

**Table 4:** Mean percent improvement and *p* values from *T*‐tests comparing the different methods against the Macintosh blade with a bougie.

	**Macintosh blade**	**Macintosh w/Bougie**	**GlideScope**	**Novel laryngoscope**

**Easy airway**				
**Novice participants**				

Improvement	22%	23%	33%	19%
*p* value	0.93		0.15	0.38

**Intermediate participants**				

Improvement	16%	11%	28%	29%
*p* value	0.72		0.00	0.00

**Experienced participants**				

Improvement	19%	9%	33%	19%
*p* value	0.33		0.07	0.69

**Easy airway**				
**Novice participants**				

Improvement	23%	12%	20%	26%
*p* value	0.79		0.87	0.73

**Intermediate participants**				

Improvement	16%	4%	19%	29%
*p* value	0.97		0.56	0.04

**Experienced participants**				

Improvement	23%	23%	11%	30%
*p* value	0.24		0.12	0.80

### 3.4. Failed Attempts

A summary of the total number of attempts, including unsuccessful attempts, is given in Table 5. During the study, investigators noted participants using the NL and the Macintosh with a bougie would occasionally lose their view of the glottis when they turned to pick up the bougie. Some participants would then incorrectly pass the bougie without looking down the scope again, such that the bougie was not visualized passing the vocal cords. Attempts at terminating due to a broken prototype were all related to a fracture at the NL’s 3D‐printed handle at the point where it meets the clear plastic tube—which was redesigned during development of the commercially available model [20]. There have been no reported breakages of the production model, the Vie Scope. Intubation failures with the GlideScope frequently concerned not being able to pass the ETT through the vocal cords even though the cords were visualized. The overall success rate is shown in Table 6.

**Table 5:** Number of attempts completed, terminated, stopped due to a broken device, and failed.

	**Macintosh laryngoscope (*n*)**	**Macintosh with bougie (*n*)**	**GlideScope (*n*)**	**Novel laryngoscope (*n*)**

*Easy airway, novice participants*				
Completed attempts	121	121	123	113
Esophageal Intubations	2	1	0	7
Broken‐device attempts	0	0	0	0
Failed attempts	1	1	0	3
Total attempts	123	123	123	123

*Easy airway, intermediate participants*				
Completed attempts	116	113	113	111
Esophageal Intubations	0	0	0	2
Broken‐device attempts	0	0	0	0
Failed attempts	1	4	4	4
Total attempts	117	117	117	117

*Easy airway, experienced participants*				
Completed attempts	54	54	54	54
Esophageal intubations	0	0	0	0
Broken‐device attempts	0	0	0	0
Failed attempts	0	0	0	0
Total attempts	54	54	54	54

*Difficult airway, novice participants*				
Completed attempts	106	95	114	110
Esophageal intubations	10	16	0	8
Broken‐device attempts	0	0	0	2
Failed attempts	7	12	9	3
Total attempts	123	123	123	123

*Difficult airway, intermediate participants*				
Completed attempts	103	93	115	96
Esophageal Intubations	3	6	0	5
Broken‐device attempts	0	0	0	4
Failed attempts	11	18	2	12
Total attempts	117	117	117	117

*Difficult airway, experienced participants*				
Completed attempts	54	52	54	52
Esophageal intubations	0	1	0	1
Broken‐device attempts	0	0	0	0
Failed attempts	0	1	0	1
Total attempts	54	54	54	54

**Table 6:** Success rate by participant type.

	Macintosh blade (%)	Macintosh w/bougie (%)	GlideScope (%)	Novel laryngoscope (%)
*Easy airway*				
Novice	98.4	98.4	100.0	91.9
Intermediate	99.1	96.6	96.6	94.9
Experienced	100.0	100.0	100.0	100.0

*Difficult airway*				
Novice	86.2	77.2	92.7	89.4
Intermediate	88.0	79.5	98.3	82.1
Experienced	100.0	96.3	100.0	96.3

## 4. Discussion

This study demonstrates that the NL is simple to learn, and repeated NL use allowed inexperienced participants​ to quickly improve intubation times in the difficult airway scenario. This is important, especially in prehospital or “out of the operating room” settings where resources such as video laryngoscopes may be unavailable or fail, or if infrequent/novice intubators must secure the patient’s airway.

The Seldinger technique using a bougie with an anterior commissure laryngoscope is perhaps an underutilized option that has been historically advocated in ENT and anesthesia literature as a rescue method when patients cannot be intubated using other common methods [17, 28, 29]. Studies in emergency medicine and anesthesia literature show it both adds to total intubation time (a median time of 10–14 s) and contributes to improved first‐pass success [5, 30]. In a recent randomized clinical trial performed on patients with difficult airways in the emergency department, the use of a bougie was associated with a significantly higher “first‐attempt” success rate than the use of an ETT and stylet (96% vs. 82%) [31]. This is consistent with the authors’ experience. In this study, there were numerous failed intubation attempts and esophageal intubations by the novice and intermediate participants, especially when the Macintosh with bougie and NL were used on the difficult airway. This seemingly conflicts with literature showing improved reliability with a Seldinger technique; however, these studies were performed with participants who would be classified as experienced. Our data reflect the fact that although a Seldinger technique may improve success in difficult airways, it is challenging and requires practice.

On the difficult airway, the NL used by novice and intermediate participants took 37.5 s compared to 32.0 s with a Macintosh blade with a bougie. There was also faster improvement (e.g., 29% with the NL vs. 4% with the Macintosh with a bougie for intermediate participants on the difficult airway). This suggests quicker learning with the NL among intermediate participants on both the easy and difficult airways. Airway professionals learning intubation generally have little to no exposure to anterior commissure laryngoscopy‐assisted intubation, which is normally performed by ear, nose, and throat surgeons and by some head and neck anesthesiologists. The NL may provide that exposure to other groups as an alternative rescue intubating tool. It also offers improved lighting and a clear viewing tube, which may be helpful to learners.

## 5. Limitations

None of the participants were given formal training with any intubation device except for watching short videos as part of this study. Trainees typically obtain hands‐on instruction with unfamiliar devices when learning a new procedural skill. This type of instruction likely would have improved the generalizability of the results. This study did not analyze the percentage of attempts that were successful for each device because the frequency of broken prototypes skews the data. This outcome is even more relevant than intubation times and would be an important target for future studies. The study did not provide an assistant to hand a bougie to the person intubating, which is different from common clinical practice in the hospital setting in particular and may have contributed to the high total number of esophageal intubations, as the user had taken their eyes away from the glottis when retrieving the bougie and did not re‐establish a glottic view when passing the bougie through the tube. Whilst many airway device studies record Cormack–Lehane grades or percentage of glottic opening (POGO), these were not used, as many of the participants were nonmedical and may have led to unnecessary confusion. The focus was on intubation success.

## 6. Conclusion

In summary, the Seldinger technique is an important skill for those who perform intubations, even infrequently or in nonideal settings. The NL, which acts as a bougie introducer, may help facilitate definitive airway management in these situations.

## Conflicts of Interest

Dr. Nilesh R. Vasan is the founder of Adroit Surgical LLC and provided the prototype intubating laryngoscopes for this study. The other authors declare no conflicts of interest.

## Author Contributions

Austin Milton: statistical analysis; manuscript and data preparation.

Rusha Patel: manuscript and data preparation and review.

Lurdes Queimado: manuscript and data preparation and review.

Price Sonkarley: conducted trials; designed the study trial; manuscript and data preparation and review.

Edward Kosik: originated the concept; designed the study trial; manuscript preparation and review.

Marvin Williams: conducted trials; manuscript and data preparation and review.

Michael Anderson: statistical analysis.

Alexis Patsias: conducted trials; manuscript and data preparation and review.

Michael Clampitt: conducted trials; manuscript and data preparation and review.

Rachel Hardy: originated the concept; design of the study trial.

Nilesh R. Vasan: originated the concept; manuscript preparation and review.

## Funding

No funding was received for this research.

## Data Availability

The entire deidentified dataset for this investigation is available upon request by contacting the corresponding author.
